# Specific Assay of Negative Strand Template to Quantify Intracellular Levels of Rhinovirus Double-Stranded RNA

**DOI:** 10.3390/mps4010013

**Published:** 2021-02-11

**Authors:** Shahina Wiehler, David Proud

**Affiliations:** Department of Physiology and Pharmacology, Snyder Institute for Chronic Diseases, Cumming School of Medicine, University of Calgary, Calgary, AB T2N 4Z6, Canada; wiehler@ucalgary.ca

**Keywords:** rhinovirus, airway epithelium, viral replication, negative strand

## Abstract

Human rhinovirus infections are a major trigger for acute exacerbations of lower airway diseases, including asthma and chronic obstructive pulmonary disease. Disease exacerbation is thought to be regulated via double-stranded RNA (dsRNA)-mediated signaling of proinflammatory and host defense responses in airway epithelial cells. Despite the central role of dsRNA in regulating host cell responses, no method for the quantitative assessment of dsRNA levels during HRV infections has been developed. Conventional RT-PCR for the negative strand template is not effective as self-priming results in apparent signals, even in the absence of primer during reverse transcription. To avoid these issues, we developed a selective assay for the negative strand template that uses a chimeric primer containing a 5′ non-viral sequence for reverse transcription and a primer using the non-viral sequence during subsequent PCR. We established that this assay avoided issues of self-priming and is strand specific, as it is unaffected even in the presence of a 1000-fold excess of positive strand. Assays in primary human airway epithelial cells showed that negative strand was detectable within 6 h of virus exposure and peaked at 18 h after virus exposure. The temporal pattern of negative strand induction mirrored that of genomic RNA but was always 1000-fold lower than positive strand, indicating that the negative strand levels regulate levels of dsRNA formation. This assay will permit relative quantification of dsRNA during studies of HRV regulation of epithelial cell function.

## 1. Introduction

Human rhinovirus (HRV) species belong to the *Enterovirus* genus of the *Picornaviradae* family. They are the dominant virus type responsible for the common cold. Importantly, HRV infections are also a major trigger of acute exacerbations in patients with lower respiratory diseases, such as asthma and chronic obstructive pulmonary disease [1]. The human airway epithelial cell (HAE) is the primary site of HRV infection and replication [2,3]. Because HRV infections do not lead to overt epithelial toxicity, either in vitro or in vivo, it is assumed that symptoms are induced as a result of alterations in epithelial cell biology that lead to increased airway inflammation. In support of this hypothesis, a number of proinflammatory cytokines and chemokines are released by HRV-infected airway epithelial cells and are also found in airway secretions during experimental or naturally acquired in vivo HRV infections [4]. Although some chemokines can be induced as a direct result of HRV binding to its receptor [5,6], the majority of proinflammatory and epithelial antiviral responses depend upon viral replication.

HRV replication requires virus entry into cells and genome release [7]. After the translation of the viral polyprotein from the positive sense RNA genome, the viral RNA polymerase generates negative strand copies of the HRV genome [8]. These negative strands then serve as templates for replication of positive strand genomes. Thus, during HRV replication, viral double-stranded RNA (dsRNA) is generated and can be recognized by pattern recognition receptors, including toll-like receptor 3 (TLR3) and the RNA helicases, retinoic acid induced gene-I (RIG-I) and melanoma differentiation associated gene 5 (mda5). The interaction of dsRNA with these receptors initiates proinflammatory and host defense gene induction [9]. Based on this concept, the majority of studies of replication-dependent responses to HRV infection have relied on the knockdown of these key pattern recognition receptors to infer a role for dsRNA in signaling [10,11,12].

A major limitation of studies of the role of dsRNA during HRV infections is that there has not been any means to quantify the relative intracellular levels of dsRNA with time. Although antibodies can be used to demonstrate the presence of dsRNA in infected cells using immunofluorescence microscopy [13], this does not permit quantification. Since negative strand RNA is created during replication and serves as the template for synthesis of multiple strands of genomic RNA, absolute quantification of negative strand would represent a reliable means of assessing levels of dsRNA. Unfortunately, it has been demonstrated for multiple single-strand positive sense viruses that standard RT-PCR cannot reliably quantify levels of negative strand RNA [14,15], particularly in the presence of excess positive strand [16]. This is due to the process of “self priming” in which signal is generated by positive strand even in the absence of primers during reverse transcription. In the current study, we sought to establish an assay to permit reliable absolute quantification of the negative strand in HRV infected primary airway epithelial cells. We provided a much-abbreviated description of this assay in a prior publication [17], but this did not include any of the validation, data and analysis included here.

## 2. Materials and Methods

### 2.1. Materials

The following reagents were purchased from the indicated suppliers: Ham’s F-12 medium, Eagle’s minimal essential medium, Hank’s balanced salt solution (HBSS), penicillin-streptomycin-amphotericin B, L-glutamine, sodium pyruvate, nonessential amino acids, gentamicin, fetal bovine serum (FBS), dNTPs, oligo(dT), random hexamers, RNase Cocktail and Maxima Reverse Transcriptase were from Invitrogen Life Technologies (Burlington, ON, Canada). Airway epithelial cell growth medium was from PromoCell GmbH (Heidelberg, Germany). Primers, probes and a GAPDH gene expression kits were from Applied Biosystems (Foster City, CA, USA); TaqMan master mix was from Roche Diagnostics (Laval, QC, Canada); All-in-one 5X All-in-one RT Master Mix was from Diamed (Mississauga, ON, Canada). All other chemicals were purchased from Sigma-Aldrich (St. Louis, MO, USA).

### 2.2. Methods

#### 2.2.1. Viral Propagation of HRV-16

Stocks of HRV-16 (family *Picornaviridae*, genus *Enterovirus*, species *Rhinovirus*) were propagated in WI-38 cells and purified by sucrose density centrifugation, as described. Viral titer was assessed using WI-38 cells, as previously described [18].

#### 2.2.2. Isolation and Culture of Human Bronchial Epithelial Cells (HBE)

HBE were obtained by protease digestion of dissected airways (main stem bronchus to fourth generation) derived from normal, non-transplanted human lungs, as previously described [19], and stored in aliquots in liquid nitrogen until used. Lungs were obtained from a tissue retrieval service (International Institute for the Advancement of Medicine, Edison, NJ, USA). Ethics approval to receive and use human lungs was obtained from the Conjoint Health Research Ethics Board of the University of Calgary. HBE were grown in submersion culture on 6-well plates in Airway Epithelial Cell Growth Medium (PromoCell Medium; PromoCell GmbH, Heidelberg, Germany). Cells were then fed every 48 h with PromoCell medium until confluent. Each “*n*” value for experimental data represents the use of cells from a different individual donor.

#### 2.2.3. Human Rhinovirus Inoculation

HBE cultures were infected with 10^5^ 50% tissue culture-infective dose (TCID_50_) of HRV-16 at 34 °C for 2 h. Cells were then washed multiple times to remove unbound virus, fresh PromoCell medium was added and cells were cultured at 34 °C for various times. Intracellular RNA was isolated for assessment of intracellular HRV levels.

#### 2.2.4. RNA Extraction and Real-Time RT-PCR

Total cellular RNA was isolated with the NucleoSpin RNA kit (Macherey-Nagel GmbH & Co, Duren, Germany), which included the homogenization of the lysates, an on-column DNase digestion and elution in RNase/DNase free water. RNA concentration and purity were determined on a NanoDrop 2000 spectrophotometer (ThermoFisher Scientific, Waltham, MA, USA). Genomic HRV-16 levels were assessed using quantitative RT-PCR using primers and a TaqMan probe directed to the 5′ untranslated region of HRV previously described [20]. A synthetic first-strand cDNA was used to generate standard curves to permit absolute quantification and data were converted to total copy number. For positive sense genomic RNA, the detection limit of the RT-PCR system corresponded to a total copy number of 10^6^.

## 3. Results

### 3.1. Rhinovirus Self-Primes in Conventional RT-PCR

**Figure 1:**
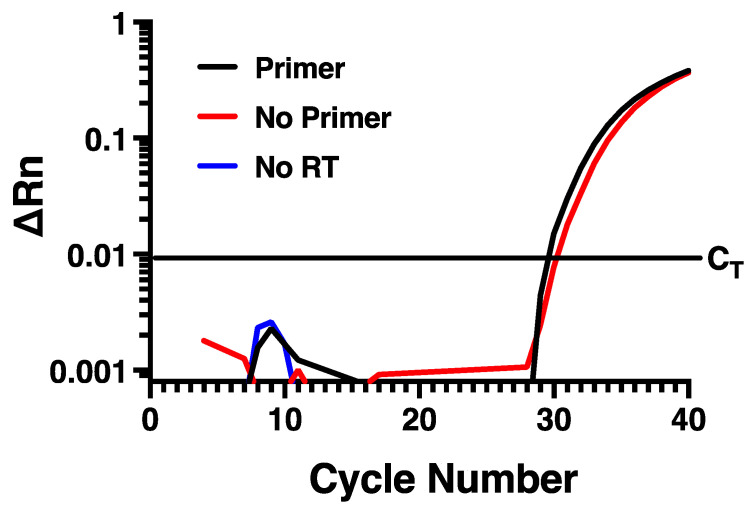
Self-priming occurs using conventional RT-PCR for negative strand. Virtually identical amplification curves are seen in the presence or absence of negative strand-specific primer during reverse transcription. Amplification is lost when reverse transcriptase is omitted. Data are representative of *n* = 4.

To determine if measuring rhinovirus negative strand RNA by standard RT-PCR approaches would be disrupted by self-priming, we designed specific forward and reverse primers and a fluorescently labeled probe (Table 1) directed within the VP1 region of the HRV-16 genome. To evaluate the potential for self-priming, total RNA harvested from infected HBE at 24 h post infection with HRV-16 was used. Reverse transcription was performed using Maxima reverse transcriptase and the reverse primer and then PCR was performed using both forward and reverse primers together with probe. As shown in Figure 1, amplification of the fluorescent signal was observed, suggesting measurement of the negative strand of the virus. Control experiments were also performed, however, in which reverse transcription reactions were also performed in which either the negative strand primer was omitted, or in which reverse transcriptase was not added. In the absence of reverse transcriptase, no amplification was observed, as would be expected. However, when the reverse transcription reaction was performed in the absence of reverse primer, the amplification curve generated was virtually identical to that generated when reverse primer was used (Figure 1). This amplification in the absence of reverse primer during reverse transcription is consistent with the phenomenon of self-priming that has been observed with several other virus types [14,15,16].

### 3.2. Tagged Primer System and Sample Clean Up Prevents Self-Priming

**Figure 2:**
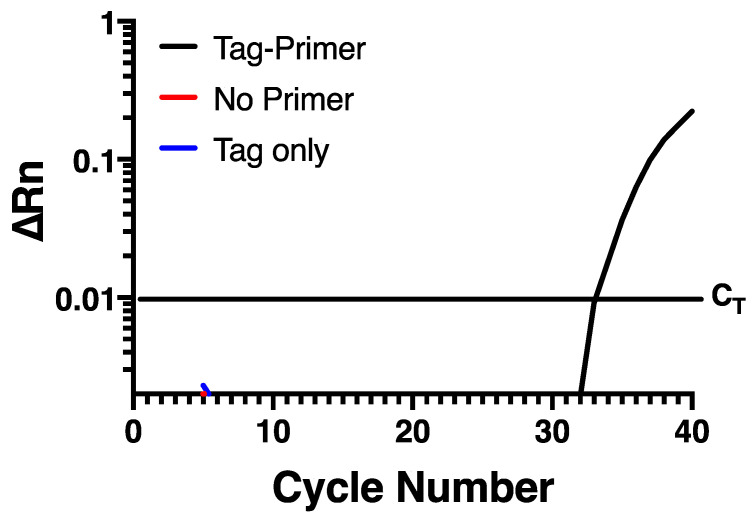
Self-priming does not occur using a chimeric primer containing a 5′ non-viral sequence. Amplification is only seen when a chimeric tagged primer is used during reverse transcription and the non-viral primer is used during PCR. Data are representative of *n* = 4.

To try and prevent self-priming, an assay was developed that uses a chimeric primer (Table 1) for reverse transcription containing a non-viral sequence at the 5′ end of the specific sequence targeting the negative strand of HRV-16. This non-viral sequence is then used as a primer to selectively amplify the negative strand specific cDNA at the PCR stage. In the current assay, the non-viral sequence was taken from within the *Escherichia coli* genome, based on the hypothesis that a bacterium may have sequences discrete from rhinovirus. A Blast search confirmed that the sequence chosen lacked any homology anywhere within the rhinovirus genome sequence. Reverse transcription of RNA from infected HBE was performed using Maxima reverse transcriptase with RNase H+ activity (Invitrogen, Burlington, ON, Canada) and the chimeric primer. To remove any potential carry over RNA, cDNA was RNase treated with an RNase cocktail (Invitrogen). The cDNA was then purified using the QIAquick PCR Purification Kit (Qiagen, Hilden, Germany). Real-time PCR was carried out using the HRV-16 negative strand-specific forward primer and probe (Table 1) and the non-viral sequence only from the chimeric primer was used as the reverse primer. When this assay was applied to RNA from infected HBE, there was a normal amplification curve when the chimeric reverse primer was used for RT-PCR, but no signal was observed either in the absence of negative primer, or in the absence of reverse transcriptase (Figure 2), indicating that self-priming was no longer an issue in this assay system.

### 3.3. Further Validation Using Excess Positive Strand

**Figure 3:**
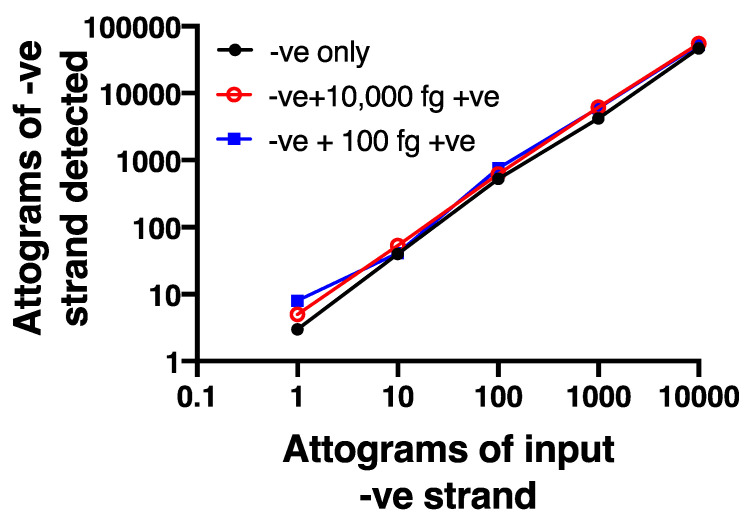
Strand specificity is observed using the chimeric primer system. A dilution curve using HPLC purified negative strand oligonucleotide is not affected even in the presence of 10- or 1000-fold excess positive strand oligonucleotide. Data are representative of *n* = 4.

To further validate the selectivity of the negative strand assay, we used synthetic, HPLC-purified negative and positive strand oligonucleotide sequences that spanned the sequences used for the assay. We first confirmed that the negative strand primer did not show any detectable signal when tested against an excess (10,000 fg) of positive strand (not shown). We also created a serial dilution curve of negative strand (1–10,000 attograms), using a first strand cDNA standard to permit absolute quantification. We then showed that adding either 100 fg (10 × the maximum amount of negative strand) or 10,000 fg (1000 × the maximum level of negative strand) of positive strand did not alter the dilution curve for negative strand (Figure 3), demonstrating no interference of excess positive strand in the assay.

### 3.4. Monitoring Positive and Negative Strand Levels in Infected Cells

**Figure 4:**
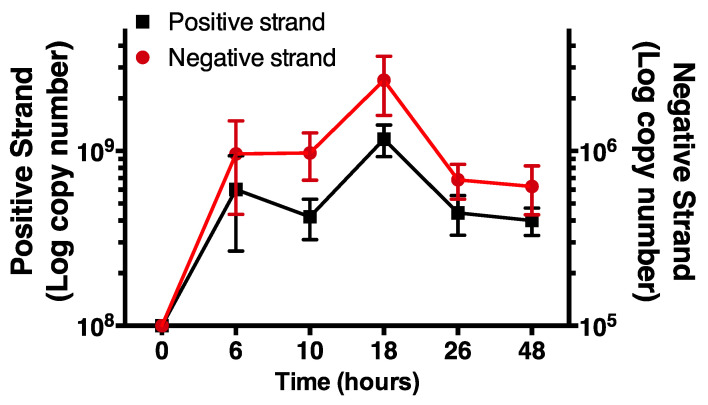
Time course of negative and positive strand signals in primary human airway epithelial cells using strand specific assays. Note the differing *y*-axis scales. Data are mean ± SEM from *n* = 4.

Having validated the selectivity of the negative strand assay, we monitored increases in time of levels of negative and positive strand HRV-16 RNA in cells infected with purified virus. Positive strand, genomic RNA was measured using a previously validated RT-PCR system, as described [20]. Cells were exposed to HRV-16 for 2 h and then washed repeatedly. At varying times after infection total cellular RNA was isolated and subjected to RT-PCR for negative strand and for positive strand. For consistency, times are shown as time from first addition of virus. Using first strand cDNA standards, each strand was expressed as absolute amounts (attograms). These values were used to calculate copy numbers of each strand. As expected, no negative strand was found in cells prior to infection but levels of negative strand RNA were increased within 6 h after virus exposure, peaking at 18 h post virus exposure. The temporal pattern of negative strand template production closely mirrored levels of positive strand genome (Figure 4). However, in all cases, levels of negative strand were approximately 1000-fold lower than those of positive strand genome.

## 4. Discussion

For all picornaviruses, including rhinoviruses, the generation of negative strand template is absolutely essential for the transcription of new genomic RNA strands [8]. The process of transcribing negative strands to genomic RNA leads to the formation of dsRNA, which is a major pathogen-associated molecular pattern that can activate proinflammatory and innate host defense responses. Although antibodies exist that can detect the presence of dsRNA in cells via imaging, these reagents suffer from a relative lack of sensitivity and do not permit any assessment of relative levels of dsRNA.

For rhinovirus, we confirmed prior studies using other virus types that have demonstrated that conventional RT-PCR targeting the negative strand does not provide strand specificity [14,15]. This has been observed for several virus families, including positive sense strand RNA viruses of the *Flaviviradae* family (hepatitis C and dengue virus) [14,15] and *Caliciviradae* family (norovirus) [16], as well as for puumala virus, a segmented negative sense strand virus of the *Hantaviridae* family [15]. Our data demonstrating that apparent negative strand could be detected, even in the absence of any primer during reverse transcription, are consistent with the previous reports of “self priming” of the RNA or of priming by exogenous nucleic acids. Moreover, it has also been reported that, when experiments using RT-PCR for negative strand were conducted using single strands generated from plasmids by in vitro transcription, that measurements of negative strand were not specific in the presence of the excess positive strand [16].

To overcome these issues, we modified a previously described strategy to design a chimeric primer for reverse transcription of negative strand in which the specific sequence targeting the negative strand of HRV-16 was extended at the 5′ end with a non-viral sequence [16]. To minimize any homology with the rhinovirus genome, we selected a non-viral sequence from the genome of *Escherichia coli*. This non-viral sequence was then used as a primer to selectively amplify the negative strand specific cDNA at the PCR stage. Using the chimeric primer, it was found that optimal results required treating the cDNA generated with an RNAse cocktail followed by the purification of the cDNA. Under these conditions, self-priming was eliminated, and the specificity of the negative strand assay was established. Further evidence of the specificity of the assay came from showing that, when purified, positive and negative strand oligonucleotides spanning the assay sequences were used, dilution curves for negative strand were unchanged even in the presence of a 1000-fold excess of positive strand oligonucleotide.

When this specific assay was used to examine both negative and positive strands in primary cultures of human airway epithelial cells grown in submersion culture, we found that the negative strand was detected within 6 h after exposure to HRV and peaked by 18 h post infection. Interestingly, the temporal patterns of negative strand levels paralleled that of levels of genomic positive strand RNA. At all time points, however, copy numbers of negative strand were approximately 1000-fold lower than positive strand copy numbers, implying that transcription of positive strand from negative strand template must be highly efficient. Our observation of a 1000:1 ratio of positive to negative strand differ from those of a previous report using an RNAse protection assay to study poliovirus in an unspecified cell line, in which a ratio of only about 70 positive strands per negative strand was reported [21]. This difference may be due to the use of poliovirus, as opposed to HRV, to the specificity and sensitivity of the different assays, or to the use of the natural cell host in our current study. We should also note that, in highly differentiated cultures of human airway epithelial cells, where viral replication is higher than in submersion culture, we also observed much lower levels of negative strand (approximately 10,000-fold) compared to positive strand numbers [17]. In any case, the presence of a 1000-fold lower level of negative strand in our current study confirms that the levels of negative strand dictate the amount of dsRNA that can be generated in epithelial cells and, thereby, regulate host immune signaling. Thus, measurements of the negative strand template will permit the assessment of relative levels of dsRNA with time during studies of host responses, either in submersion culture or in epithelial cells grown at the air–liquid interface.

## Figures and Tables

**Table 1:** Primer and probe sequences used for the negative strand HRV-16 RT-PCR reactions.

Name	Purpose of Oligonucleotide	Sequence
Neg RT primer	Standard Negative RT/PCR primer	5′-GGCAGCATGGGCAACCT-3′
For HRV16 primer	Real-time PCR Forward Primer	5′-TGCTGATGCAATACTCAAAAAGG-3′
Neg-Tag-cDNA	Chimeric RT primer	5′-ATCAGCGATGCCGAACGTATGGCAGCATGGGCAACCT-3′
Tag-Rev	Real-time PCR Reverse Primer	5′-ATCAGCGATGCCGAACGTAT-3′
Probe	Real-time PCR Probe	5′FAM-TGAAAAGCGAGGGA-MGB3′

Underlined nucleotides denote non-viral tag sequences.

## Data Availability

Data sharing not applicable.
